# Reduced irrigation decreases the optimal nitrogen threshold of greenhouse tomatoes by altering gas exchange and stomatal morphology

**DOI:** 10.3389/fpls.2026.1897205

**Published:** 2026-08-19

**Authors:** Lingxiang Zheng, Yu Zhao, Binbin Han, Danyang Ma, Junsheng Lu, Zhijie Chang, Yanwei Fan

**Affiliations:** 1 Pingliang Municipal Water Resources Center, Pingliang, Gansu, China; 2 Key Laboratory of Agricultural Soil and Water Engineering in Arid and Semiarid Areas, Ministry of Education, Northwest A&F University, Yangling, Shaani, China; 3 College of Civil and Hydraulic Engineering, Lanzhou University of Technology, Lanzhou, China

**Keywords:** irrigation, nitrogen application rate, Rubisco, stomata, tomato

## Abstract

In arid regions, agricultural water shortages and excessive nitrogen fertilization severely restrict the sustainable development of the tomato industry. To elucidate the effects of water-nitrogen interactions on the yield and photosynthetic physiological characteristics of tomatoes, a two-year (2024 and 2025) split-plot greenhouse experiment was conducted. The main plots evaluated two irrigation lower limits at 75% (W1) and 65% (W2) of field capacity (FC), and the subplots consisted of four nitrogen application rates: 0, 150, 300, and 450 kg ha^-1^ (N0, N1, N2, and N3). The results indicated that: (1) The responses of fruit yield, aboveground biomass, and water productivity (WP) to the nitrogen application rate all exhibited quadratic parabolic trends. Under W2, the N1 and N2 eliminated the yield discrepancy with W1, and increased WP and agronomic nitrogen use efficiency by 9.9%–11.7% and 30.4%–35.1%, respectively. Compared to N0, the N1 and N2 increased fruit yield by 33.3%–34.9% and 52.8%–53.1%, respectively. (2) W2 caused the optimal nitrogen thresholds to shift toward lower nitrogen levels. The optimal nitrogen rates for fruit yield and WP under W1 were 344.2 and 346.1 kg ha^-1^, respectively, which decreased to 279.9 and 264.8 kg ha^-1^ under reduced irrigation (W2). Concurrently, the optimal nitrogen rate for net photosynthetic rate (*P*_n_) declined from 394.6 kg ha^-1^ under full irrigation to 300.1 kg ha^-1^. (3) A random forest model demonstrated that the SPAD value, intrinsic water use efficiency, Rubisco activity, and stomatal width were the core factors determining *P*_n_. Moderate nitrogen application (W2N2) induced a “high-density and small-aperture” stomatal morphological trait and significantly enhanced Rubisco activity. (4) Under W2, the N3 led to a significant accumulation of malondialdehyde concentration, accompanied by marked decreases in the SPAD value and Rubisco activity, ultimately causing a decline in *P*_n_ and a significant yield reduction. In conclusion, setting the irrigation lower limit to 65%FC combined with a nitrogen rate of 265–280 kg ha^-1^ constitutes a precision management strategy for synergistically achieving water conservation, stable yields, and high water-fertilizer use efficiency in greenhouse tomatoes.

## 1 Introduction

Tomato is one of the most important vegetable crops globally, playing an irreplaceable role in fulfilling human dietary nutritional needs and promoting agricultural economic development (Chen et al., 2013; Maureira et al., 2022). Greenhouses provide a controllable growing environment for tomatoes, completely isolating them from natural rainfall; therefore, artificial irrigation serves as the sole pathway for crop water acquisition (Wu et al., 2021). Given this absolute reliance on irrigation, in arid and semi-arid regions, agricultural water shortages have become the core bottleneck restricting the sustainable development of the greenhouse industry (Chen et al., 2013). To pursue high yields, traditional greenhouse cultivation often relies on excessive water and fertilizer inputs (Sun et al., 2024). This management practice not only severely wastes precious water resources, but the excessive application of nitrogen fertilizer also fails to deliver a proportional increase in yield (Du et al., 2024). On the contrary, excessive nitrogen application triggers severe ecological and environmental issues (Chen et al., 2020). For instance, nitrogen generates greenhouse gases through ammonia volatilization and denitrification, exacerbating air pollution; simultaneously, surplus chemical fertilizers remaining in the soil lead to soil acidification and secondary salinization, severely degrading soil quality (Wu et al., 2019). Therefore, formulating precise and integrated water and nitrogen management strategies within greenhouse environments devoid of precipitation interference, to achieve water conservation, stable yields, and environmental sustainability, is an urgent challenge currently facing modern agriculture.

Deficit irrigation is an effective strategy to address agricultural water scarcity (Wang et al., 2023). Although moderate water deficit can improve water productivity and fruit quality, improper management inevitably leads to a decline in yield (Yu et al., 2020). To mitigate this deficit-induced yield loss, rational nitrogen management is crucial, as nitrogen directly dictates the physiological adaptability of crops to moisture constraints (Johora et al., 2026). Under optimal application, nitrogen can exert a “fertilizer-driven water regulation” compensatory effect, effectively alleviating the yield penalties caused by water stress (Agami et al., 2018). However, successfully harnessing this compensatory effect requires a delicate balance, because soil moisture conditions fundamentally restrict nitrogen availability and crop tolerance. Under reduced irrigation, decreased soil moisture severely limits the dissolution and transport of nitrogen (He et al., 2024). Consequently, if the nitrogen application rate exceeds a critical threshold under such water-limited conditions, the unabsorbed fertilizer not only fails to promote growth but sharply increases the electrical conductivity of the soil solution. This exacerbates osmotic stress in the root zone, turning the intended compensation into a severe water-fertilizer antagonism (He et al., 2024; Zhao et al., 2026). Because water stress simultaneously increases the crop’s reliance on nitrogen for adaptation while lowering the safe threshold for nitrogen application, the synergistic or antagonistic mechanisms between varying irrigation lower limits and gradient nitrogen application rates remain highly complex. Particularly, how the optimal nitrogen thresholds for maximizing tomato yield dynamically shift under varying degrees of water deficit requires further systematic exploration.

A comprehensive understanding of this water-fertilizer interactive effect necessitates an in-depth exploration of the micro-physiological mechanisms that link agronomic inputs to final yield formation (Zhou et al., 2020; He et al., 2024; Johora et al., 2026). Stomata act as the pivotal gateways for carbon dioxide uptake and water loss between plants and the external environment, and their density and width directly regulate leaf stomatal conductance, thereby determining the balance between the net photosynthetic rate and the transpiration rate (Zheng et al., 2020). Simultaneously, the activity of key photosynthetic enzymes within mesophyll cells (e.g., ribulose-1,5-bisphosphate carboxylase/oxygenase, Rubisco) constitutes the biochemical foundation of carbon assimilation (Halpern et al., 2019; Chang et al., 2025). The supply status of water and nitrogen also significantly impacts plant cell membrane stability (Saneoka et al., 2004). When crops are subjected to drought or to salt-induced osmotic stress triggered by excessive nitrogen application, a massive burst of reactive oxygen species (ROS) occurs within the leaves, leading to the rapid accumulation of lipid peroxidation products (e.g., malondialdehyde, MDA) (Saneoka et al., 2004; Cao et al., 2019). Such oxidative damage directly compromises chloroplast structural integrity, resulting in chlorophyll degradation and the inactivation of photosynthetic enzymes, ultimately forcing stomatal closure and impairing the photosynthetic capacity (Sahu and Kar, 2018; Wei et al., 2019).

Although previous studies have investigated the impacts of deficit irrigation alone on tomato yield and water consumption characteristics, or individually evaluated the responses of specific physiological indicators, comprehensive research that systematically integrates irrigation levels and nitrogen application rates with stomatal traits, photosynthetic enzyme activity, cellular oxidative damage, and ultimate resource use efficiency remains scarce (Coyago-Cruz et al., 2019; Zhou et al., 2025; Jiao et al., 2026). This mechanistic knowledge gap restricts our ability to explain why an appropriate nitrogen application can maintain high yields under reduced irrigation, and why the combination of high nitrogen and water deficit triggers a decline in plant physiological functions. Elucidating these intrinsic micro-level drivers is of profound scientific significance for optimizing the precise water and fertilizer management of greenhouse crops.

To address the aforementioned knowledge gap, a two-year greenhouse experiment was conducted in this study. We systematically evaluated the comprehensive impacts of different irrigation levels and nitrogen application rates on tomatoes. We proposed the following scientific hypotheses: (i) the optimal nitrogen threshold for maximizing tomato yield and water productivity will shift toward lower nitrogen levels as the irrigation level decreases; (ii) under reduced irrigation, an appropriate nitrogen application rate can secure carbon assimilation capacity by optimizing stomatal structure and maintaining Rubisco activity, thereby narrowing the yield gap with full irrigation; and (iii) the combination of reduced irrigation and excessive nitrogen application induces severe combined stress, leading to substantial MDA accumulation and impaired photosynthetic function, ultimately causing a significant reduction in fruit yield.

## 2 Materials and methods

### 2.1 Experiment site

The field experiments were conducted in 2024 and 2025 in a greenhouse located in Yuzhong County (104°09′ E, 35°56′ N, altitude 1749 m). The greenhouse was 60 m long and 8 m wide. The experimental site is situated in a typical semi-arid climate zone, characterized by an average annual evaporation of 1450 mm, an average annual precipitation of 327 mm, and an average annual temperature of 7.6 °C. The accumulated temperature ≥ 0 °C is 3244 °C, the accumulated temperature ≥ 10 °C is 2479 °C, and the annual sunshine duration ranges from 1626 to 2666 h. The soil at the site is loessial soil. Within the 0–20 cm topsoil layer, the soil bulk density is 1.16 g cm^-3^, the total nitrogen content is 1.35 g kg^-1^, the available potassium content is 325 mg kg^-1^, the available phosphorus content is 28 mg kg^-1^, and the field capacity is 0.28 cm^3^ cm^-3^.

### 2.2 Experiment design

This study adopted a split-plot design consisting of irrigation levels as the main plots and nitrogen treatments as the subplots. The irrigation lower limits were set at 75% (W1) and 65% (W2) of the field capacity (FC). Four nitrogen application levels were established: 0, 150, 300, and 450 kg ha^-1^. Each treatment consisted of three independent plots, with each plot representing a single replicate (totaling 24 experimental subplots). The nitrogen fertilizer was applied across the seedling, flowering and fruit-setting, fruit expansion, and mature harvesting stages at proportions of 10%, 20%, 50%, and 20%, respectively. P_2_O_5_ and K_2_O were applied as base fertilizers at rates of 120 kg ha^-1^ and 300 kg ha^-1^, respectively. The chemical fertilizers used were urea, calcium superphosphate, and potassium sulfate.

During the experiment, the volumetric soil moisture content within the 0–60 cm soil layer was monitored regularly using a portable soil moisture monitoring system (Diviner 2000, Sentek Pty Ltd, Australia). When the soil moisture dropped to the designated lower limits, irrigation was immediately initiated to replenish the soil water to the corresponding upper limits. To ensure experimental consistency, the soil moisture control intervals for the two irrigation treatments were identically set to a 10% FC range: 75%–85% FC for the W1 treatment and 65%–75% FC for the W2 treatment. The specific water amount for each plot was strictly controlled and recorded using water meters. Based on the uniform moisture replenishment interval (10% FC), the single irrigation quota was maintained within a range of 15–18 mm for each event in both treatments. Furthermore, the irrigation frequency varied dynamically, driven by the varying evapotranspiration rates across different growth stages. A unified dynamic irrigation schedule was applied across all treatments. Specifically, during the seedling stage, slower water consumption extended the irrigation intervals to 7 to 10 days. During the peak water-demanding fruit expansion stage, rapid water consumption shortened the intervals to 4 to 6 days. Subsequently, during the late maturation stage, the intervals were extended again to 10 to 12 days. As a result, the total seasonal irrigation amounts applied were 305.7 mm and 256.8 mm in 2024, and 295.6 mm and 248.3 mm in 2025 for the W1 and W2 treatments, respectively.

The tomato cultivar ‘Jinpeng No. 1’ was used in this study, planted at a row spacing of 0.5 m and a plant spacing of 0.35 m. Transplanting was conducted in early March of each year. Immediately after transplanting, all plots were uniformly irrigated with 20 mm of water. The entire soil surface was covered with plastic mulch to minimize soil evaporation. To prevent the lateral movement of water and nutrients between adjacent plots, plastic films were buried to a depth of 1 m between the plots to serve as isolation barriers. Irrigation and fertilization were administered using a drip irrigation system under the plastic mulch. The drip tapes had an emitter spacing of 0.3 m, an emitter flow rate of 2.4 L h^-1^, and a rated operating pressure of 0.1 MPa. During the experimental period, except for the irrigation and nitrogen treatments, all other agronomic management practices, such as pollination, pruning, and pest control, were identical across all plots.

### 2.3 Measurement indicators and methods

#### 2.3.1 Fruit yield and biomass

At the maturity stage, 20 tomato plants were randomly selected from each of the three replicate plots per treatment to determine the fruit yield (totaling 60 sampled plants per treatment). To determine the aboveground biomass, three randomly selected plants from each of the three replicate plots (totaling 9 sampled plants per treatment) were heated at 105 °C for 30 min to deactivate enzymes, and subsequently oven-dried to a constant weight at 75 °C.

#### 2.3.2 Water productivity and agronomic nitrogen utilization efficiency

The estimation of crop water use was conducted using the following field water balance (Equation 1) (Allen et al., 1998):

(1) ET=P+I+K−D−R±ΔW

Where ET represents crop water use, P stands for precipitation, I denotes the applied irrigation, K is the groundwater contribution, D represents deep percolation, R denotes surface runoff, and ΔW signifies the fluctuation in soil water storage in the 0–60 cm soil profile (all expressed in mm). Given that the experiment was conducted in a greenhouse without rainfall, P was zero. Due to the flat terrain and the use of under-mulch drip irrigation, surface runoff (R) was assumed to be zero. Furthermore, lysimeters with leakage collection vessels were installed 0.6 m beneath the soil surface in the control plots to collect drainage water. No drainage was observed from the lysimeters, so D was considered 0. The groundwater table at the experimental site was below 6.0 m, rendering it inaccessible for crop utilization; therefore, K was set to 0. Consequently, Consequently, Equation 1 can be simplified as Equation 2 (Chen et al., 2013):

(2) ET=I±ΔW

Water productivity (WP) and agronomic nitrogen use efficiency (ANUE) were calculated based on the tomato yield, cumulative water consumption, and nitrogen application rate using the following (Equations 3, 4):

(3) $$WP=\frac{Y}{10ET}$$

(4) $$ANUE=\frac{Y-Y_{non}}{F_{N}}$$

Where WP is the water productivity (kg m^-3^); ANUE is the agronomic nitrogen use efficiency (kg kg^-1^); Y is the fruit yield (kg ha^-1^); Y_non_ is the fruit yield under the non-nitrogen treatment (kg ha^-1^); and F_N_ is the nitrogen application rate (kg ha^-1^).

#### 2.3.3 Leaf gas exchange, leaf stomatal traits and SPAD

During the fruit expansion stage, gas exchange measurements were conducted between 09:00 and 11:00 on clear days. The fifth fully expanded, healthy leaf from the apex of three randomly selected tomato plants in each plot was selected. A portable photosynthesis system (LI-6800; LI-COR Biosciences, Lincoln, NE, USA) equipped with an internal red/blue LED light source was used to measure the net photosynthetic rate (*P*_n_), transpiration rate (*T*_r_), and stomatal conductance (*G*_s_). To ensure the consistency of the measurement standards, the environmental parameters within the leaf chamber were uniformly controlled: the photosynthetically active radiation (PAR) was set to 1200 μmol m^-2^ s^-1^, the reference CO_2_ concentration was maintained at 400 μmol mol^-1^, the leaf temperature was set to 25°C, and the relative humidity was controlled at 60%. The selected tomato leaves completely covered the standard leaf chamber area, so no area correction was required. Data was recorded once the system readings stabilized. The intrinsic water use efficiency (*WUE*_i_) was calculated as the ratio of *P*_n_ to *G*_s_. As described by Chang et al. (2025), concurrently with the measurement of leaf gas exchange parameters, the nail polish impression method was employed to determine the stomatal density and stomatal width of the identical leaves used for *P*_n_ determination. Stomatal impressions were collected from both the adaxial and abaxial surfaces of the leaves, and the mean values of the two surfaces were calculated for subsequent analysis. In this study, stomatal width was defined and measured as the total width of the stomatal complex (i.e., the maximum distance between the outer edges of the two guard cells, including the stomatal pore), representing the potential opening limit of the stomata for each treatment. Simultaneously, the relative chlorophyll content of these same leaves was measured using a SPAD chlorophyll meter (SPAD-502, Japan). All measurements were performed with three replicates.

#### 2.3.4 Leaf enzyme activity and concentration

During the fruit enlargement stage, the identical leaves used for the leaf gas exchange measurements were also collected to determine the leaf Rubisco activity and malondialdehyde (MDA) concentration (n = 3). The Rubisco activity and MDA concentration were determined using commercial enzyme-linked immunosorbent assay (ELISA) kits (Shanghai Lengton Bioscience Co., Ltd., Shanghai, China) following the methods described by Liu et al. (2022) and Awad et al. (2011). All specific operations were performed strictly in accordance with the manufacturer’s instructions.

### 2.4 Statistical analyses

A three-way analysis of variance (ANOVA) was employed to evaluate the effects of irrigation (I), nitrogen application rate (N), year (Y), and their interactions on fruit yield, biomass, WP, ANUE, leaf gas exchange parameters, stomatal parameters, photosynthetic enzyme activity, and MDA concentration. Based on the split-plot design, a one-way ANOVA was conducted separately for the main plots and subplots to compare treatment means at a significance level of 0.05. Linear regression models were used to analyze the relationships between the various indicators and the nitrogen application rate. Furthermore, a random forest model was constructed using the “randomForest” package in R to determine the relative importance of key factors affecting tomato *P*_n_ under different irrigation and nitrogen application rates. The Random Forest (RF) model was constructed using the “randomForest” and “rfPermute packages” in R to evaluate variable importance. The number of decision trees (ntree) was set to 2000, and the statistical significance of variable importance (%IncMSE) was assessed via a permutation test with 299 replications (nrep = 299). The model’s predictive reliability was validated using Out-of-Bag (OOB) estimation, evaluated by R-squared (R^2^) and Normalized Root Mean Squared Error (NRMSE). All statistical analyses were performed using SPSS version 21.0 (SPSS Inc., Chicago, IL, USA), and data visualization was conducted using Origin 2026 (OriginLab Corp., Northampton, MA, USA).

## 3 Results

### 3.1 Fruit yield and biomass

**Figure 1:**
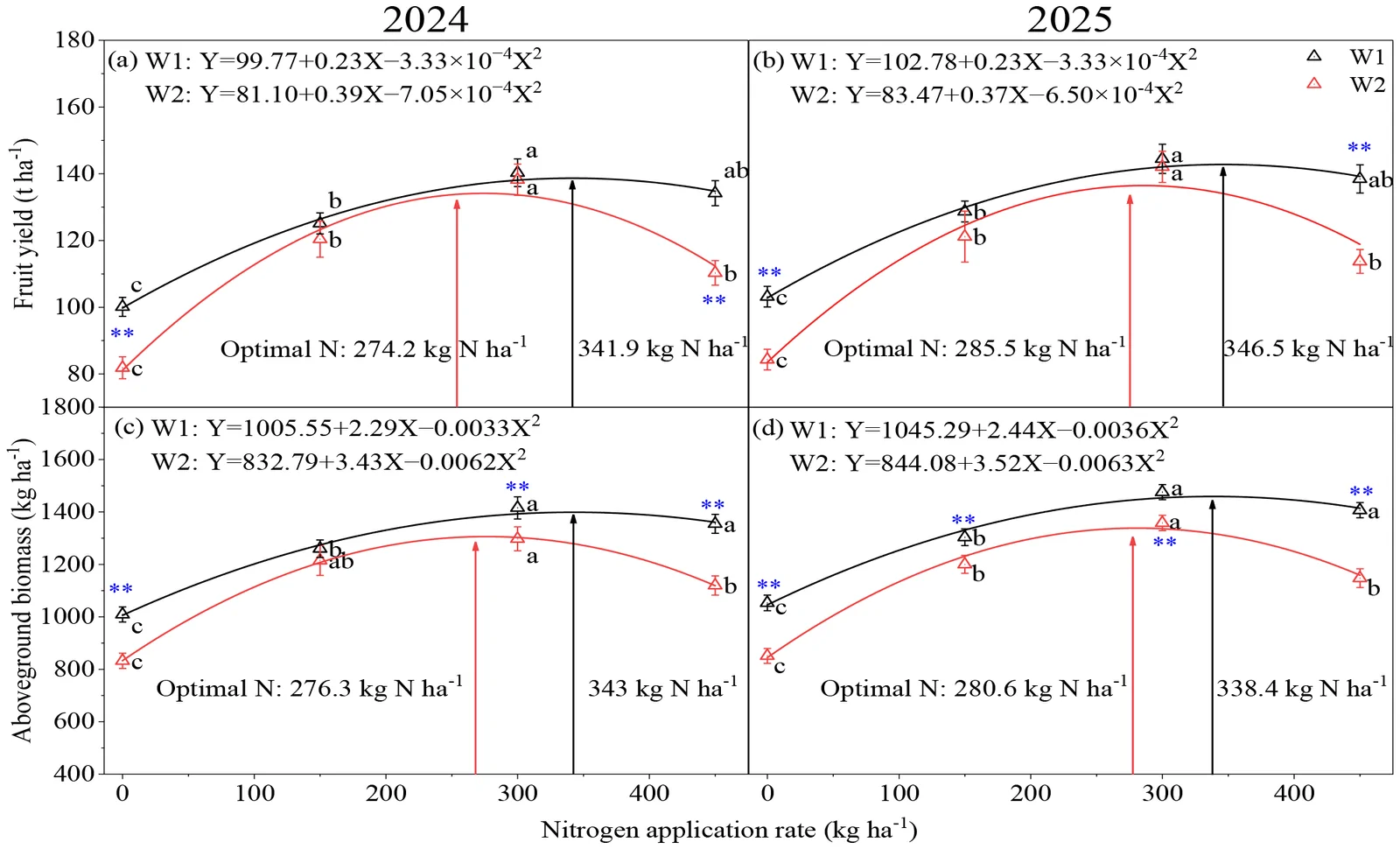
Effects of nitrogen fertilizer application rates on fruit yield **(a, b)** and aboveground biomass **(c, d)** under different irrigation levels. W1 and W2 represent full irrigation and reduced irrigation, respectively. Different lowercase letters indicate significant differences among treatments within the same irrigation level at the 0.05 level (*P* < 0.05). **indicates significant differences among different irrigation levels under the same nitrogen rate at the 0.01 level (*P* < 0.01).

Irrigation, nitrogen application rate, year, and the irrigation × nitrogen (I × N) significantly affected fruit yield and aboveground biomass (all *P* < 0.001; **Table 1**). Compared with W1, W2 decreased the fruit yield by 9.8%–10.4% (**Figure 1**). Compared with N0, the N1, N2, and N3 increased the fruit yield by 33.3%–34.9%, 52.8%–53.1%, and 34.3%–34.5%, respectively. Furthermore, under different irrigation levels, the response of fruit yield to the increasing nitrogen application rate exhibited a quadratic parabolic trend, initially increasing and then decreasing, indicating the existence of an optimal nitrogen application rate. Across 2024 and 2025, the average optimal nitrogen application rates for fruit yield under W1 and W2 were 344.2 kg ha^-1^ and 279.9 kg ha^-1^, respectively. Under the N0 and N3, W2 significantly reduced the fruit yield compared to W1 (all *P* < 0.001). However, under the N1 and N2, there was no significant difference in fruit yield between W1 and W2 (*P*>0.05).

**Table 1:** Three-way ANOVA of the effects of irrigation, nitrogen fertilizer application rates and year on tomato indicators.

Indicator	Irrigation	Nitrogen	Year	I×N	I×Y	N×Y	I×N×Y
Fruit yield	*P* < 0.001	*P* < 0.001	*P* = 0.013	*P* < 0.001	*P* = 0.642	*P* = 0.938	*P* = 0.983
Aboveground biomass	*P* < 0.001	*P* < 0.001	*P* = 0.001	*P* < 0.001	*P* = 0.204	*P* = 0.471	*P* = 0.791
WP	*P* < 0.001	*P* < 0.001	*P* = 0.212	*P* < 0.001	*P* = 0.404	*P* = 0.559	*P* = 0.505
ANUE	*P* < 0.001	*P* < 0.001	*P* = 0.905	*P* < 0.001	*P* = 0.747	*P* = 0.894	*P* = 0.892
*P*_n_	*P* < 0.001	*P* < 0.001	*P* = 0.589	*P* < 0.001	*P* = 0.311	*P* = 0.778	*P* = 0.889
*T*_r_	*P* < 0.001	*P* < 0.001	*P* = 0.876	*P* < 0.001	*P* = 0.087	*P* = 0.332	*P* = 0.190
*G*_s_	*P* < 0.001	*P* < 0.001	*P* = 0.013	*P* = 0.001	*P* = 0.446	*P* = 0.886	*P* = 0.751
*WUE*_i_	*P* = 0.001	*P* < 0.001	*P* < 0.001	*P* < 0.001	*P* = 0.317	*P* = 0.794	*P* = 0.222
SPAD	*P* < 0.001	*P* < 0.001	*P* = 0.411	*P* = 0.050	*P* = 0.712	*P* = 0.945	*P* = 0.979
Stomatal density	*P* < 0.001	*P* < 0.001	*P* = 0.045	*P* < 0.001	*P* = 0.775	*P* = 0.359	*P* = 0.412
Stomatal width	*P* < 0.001	*P* < 0.001	*P* = 1.000	*P* < 0.001	*P* = 0.030	*P* = 0.416	*P* = 0.450
Rubisco activity	*P* < 0.001	*P* < 0.001	*P* = 0.529	*P* = 0.001	*P* = 0.614	*P* = 0.975	*P* = 0.948
MDA concentration	*P* < 0.001	*P* < 0.001	*P* = 0.032	*P* < 0.001	*P* = 0.280	*P* = 0.636	*P* = 0.753

WP, ANUE, *P*_n_, *T*_r_, *G*_s_, and *WUE*_i_ represent water productivity, agronomic nitrogen use efficiency, net photosynthetic rate, transpiration rate, stomatal conductance, and intrinsic water use efficiency, respectively.

Compared with W1, W2 decreased the aboveground biomass by 11.4%–13.0%. Compared with N0, the N1, N2, and N3 increased the aboveground biomass by 31.5%–34.4%, 47.3%–48.8%, and 34.1%–34.4%, respectively. Furthermore, unlike the response of fruit yield, W2 significantly reduced the aboveground biomass compared to W1 at the N0, N2, and N3 levels (all *P* < 0.01). The aboveground biomass exhibited a parabolic trend, initially increasing and then decreasing, with the increment of the nitrogen application rate. Across the two years, the average optimal nitrogen application rates for aboveground biomass under W1 and W2 were 340.7 kg ha^-1^ and 278.5 kg ha^-1^, respectively.

### 3.2 Water productivity and agronomic nitrogen utilization efficiency

**Figure 2:**
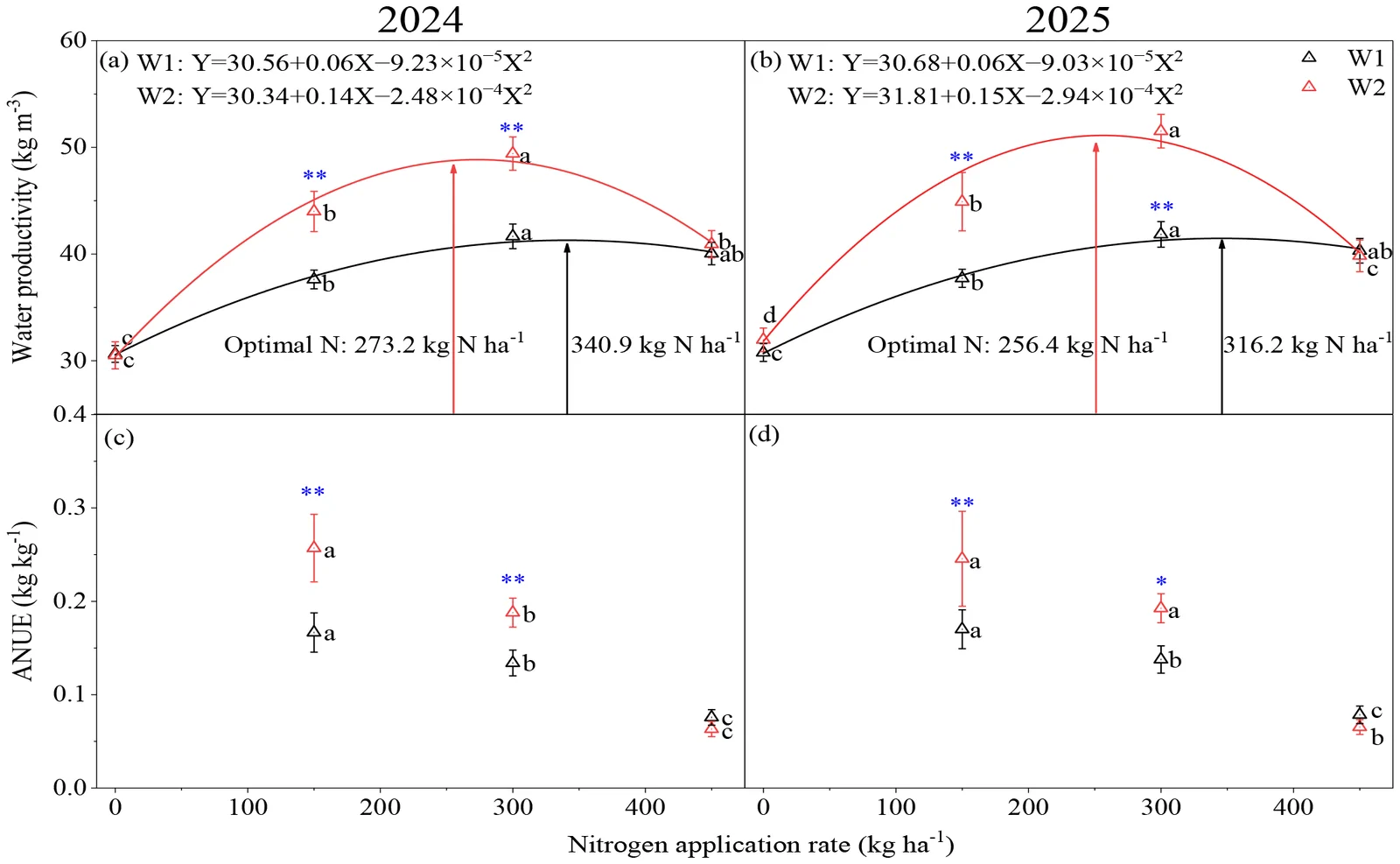
Effects of nitrogen fertilizer application rates on tomato water productivity **(a, b)** and agronomic nitrogen use efficiency **(c, d)** under different irrigation levels. ANUE represents agronomic nitrogen use efficiency. W1 and W2 represent full irrigation and reduced irrigation, respectively. Different lowercase letters indicate significant differences among treatments within the same irrigation level at the 0.05 level (*P* < 0.05). * and ** indicate significant differences among irrigation levels under the same nitrogen rate at the 0.05 and 0.01 levels, respectively.

Irrigation, nitrogen application rate, and their interactive effects significantly altered water productivity (WP) and agronomic nitrogen use efficiency (ANUE) (all *P* < 0.001; **Table 1**). Compared with W1, W2 increased WP by 9.9%–11.7% (all *P* < 0.001; **Figure 2**). Compared with N0, the N1, N2, and N3 treatments increased WP by 33.5%–35.1% (all *P* < 0.001), 49.0%–52.7% (all *P* < 0.001), and 31.1% (*P* = 0.001)–32.4% (*P* < 0.001), respectively. Under the N1 and N2 levels, W2 significantly increased WP compared to W1. The average optimal nitrogen application rates for WP under W1 and W2 were 346.1 kg ha^-1^ and 264.8 kg ha^-1^, respectively. Furthermore, compared with W1, W2 increased ANUE by 30.4%–35.1%. Under different irrigation levels, ANUE gradually decreased with the increment of the nitrogen application rate, reaching its minimum under the N3 treatment. Under the N1 and N2 levels, W2 significantly increased ANUE compared to W1.

### 3.3 Gas exchange and SPAD

**Figure 3:**
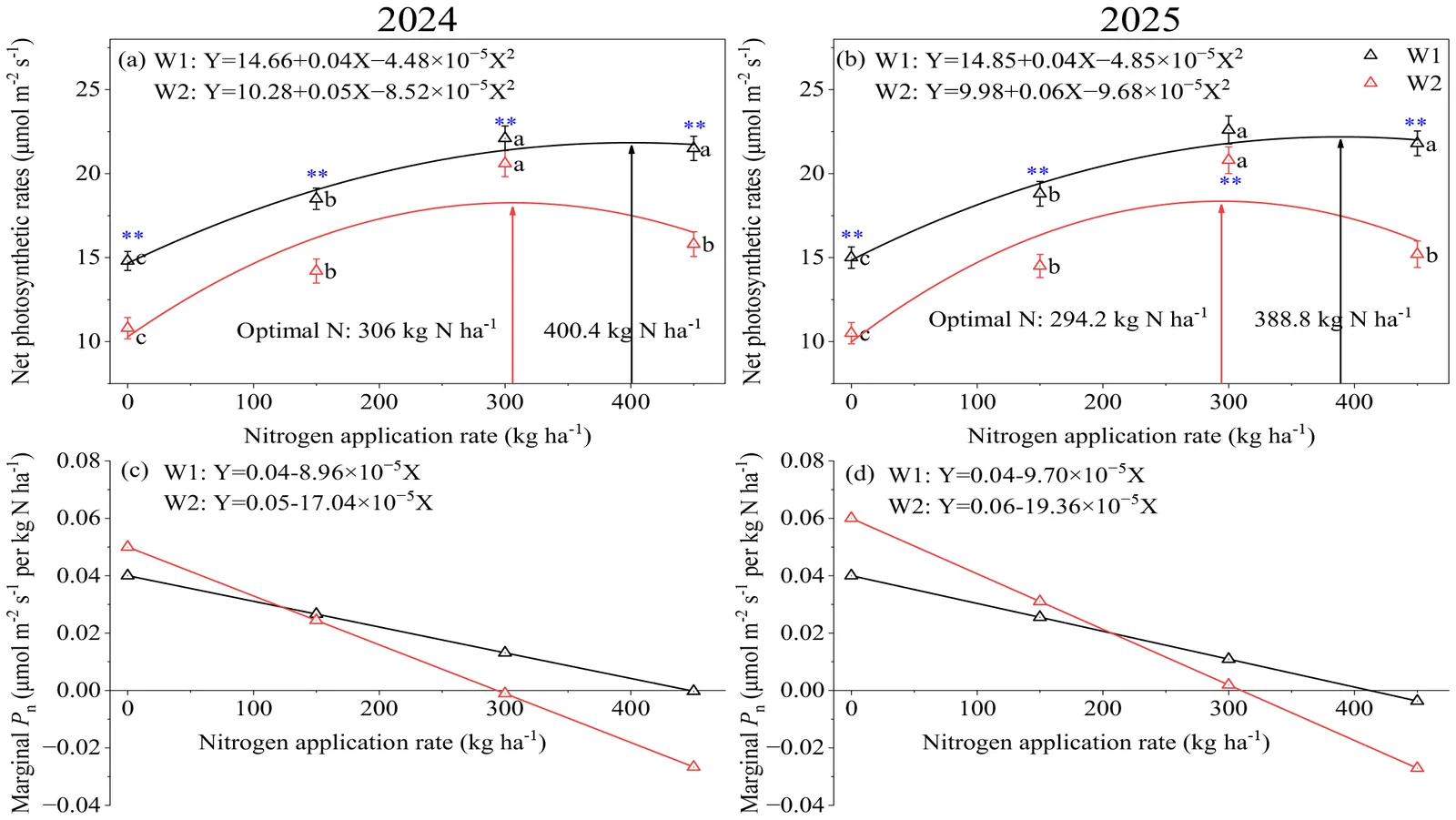
Effects of nitrogen fertilizer application rates on net photosynthetic rate **(a, b)** and marginal net photosynthetic rate **(c, d)** of tomato under different irrigation levels. *P*_n_ represents net photosynthetic rate. W1 and W2 represent full irrigation and reduced irrigation, respectively. Different lowercase letters indicate significant differences among treatments within the same irrigation level at the 0.05 level (*P* < 0.05). **indicates significant differences among different irrigation levels under the same nitrogen rate at the 0.01 level (*P* < 0.01).

The study revealed that irrigation, nitrogen application rate, and their interaction significantly affected *P*_n_, *T*_r_, *G*_s_, and *WUE*_i_ (all *P ≤* 0.001), with the year also exerting a significant effect on *G*_s_ (*P* = 0.013) and *WUE*_i_ (*P* < 0.001) (**Table 1**). Compared with W1, W2 decreased *P*_n_ by 20.2%–22.0% (**Figures 3a, b**). Compared with N0, the N1, N2, and N3 treatments increased *P*_n_ by 27.7%–30.6%, 66.8%–70.2%, and 45.1%–45.7%, respectively (all *P* < 0.001). Across all nitrogen application rates, W2 significantly reduced *P*_n_ compared to W1. The average optimal nitrogen application rates under W1 and W2 were 394.6 kg ha^-1^ and 300.1 kg ha^-1^, respectively. Furthermore, taking the derivative of the regression equation between *P*_n_ and the nitrogen application rate revealed that the marginal *P*_n_ decreased linearly with the increment of the nitrogen application rate. Notably, the marginal *P*_n_ under W2 declined more rapidly than that under W1 (**Figures 3c, d**). This indicates that under W2, the response of leaf *P*_n_ to changes in the nitrogen application rate was more pronounced.

**Figure 4:**
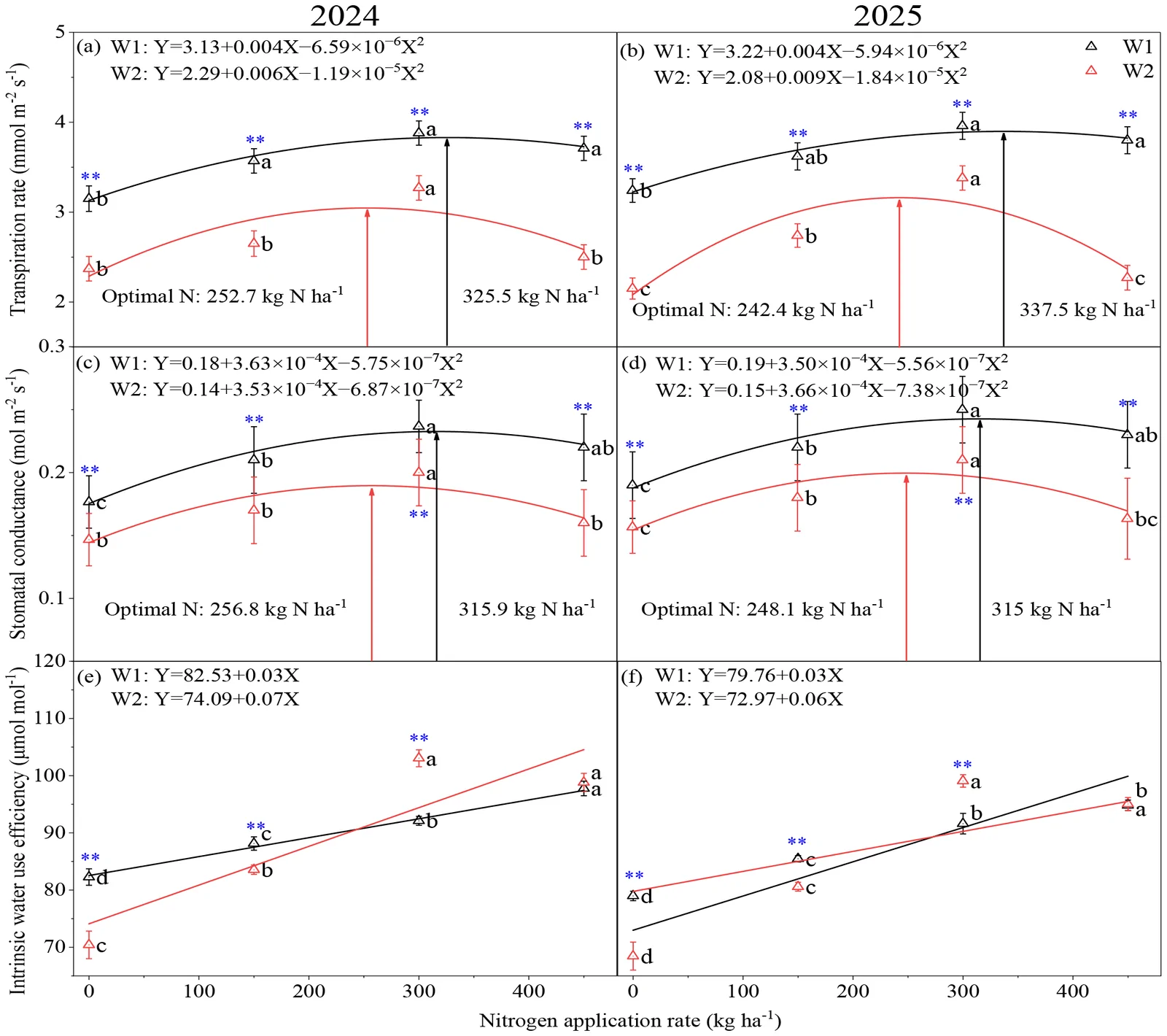
Effects of nitrogen fertilizer application rates on transpiration rate **(a, b)**, stomatal conductance **(c, d)**, and intrinsic water use efficiency **(e, f)** of tomato under different irrigation levels. W1 and W2 represent full irrigation and reduced irrigation, respectively. Different lowercase letters indicate significant differences among treatments within the same irrigation level at the 0.05 level (*P* < 0.05). **indicates significant differences among different irrigation levels under the same nitrogen rate at the 0.01 level (*P* < 0.01).

Under different nitrogen application rates, W2 significantly reduced *T*_r_ and *G*_s_ compared to W1 (**Figure 4**). Under different irrigation levels, both *T*_r_ and *G*_s_ exhibited a parabolic trend, initially increasing and then decreasing, with increasing nitrogen application rates. The average optimal nitrogen application rates for *T*_r_ under W1 and W2 were 331.5 kg ha^-1^ and 247.6 kg ha^-1^, respectively, whereas those for *G*_s_ were 315.5 kg ha^-1^ and 252.5 kg ha^-1^, respectively. In contrast, *WUE*_i_ increased linearly with increasing nitrogen application rates. At nitrogen application rates exceeding 300 kg ha^-1^, *WUE*_i_ under W2 was higher than that under W1, a difference that was particularly significant under the N2 treatment (*P* < 0.01).

**Figure 5:**
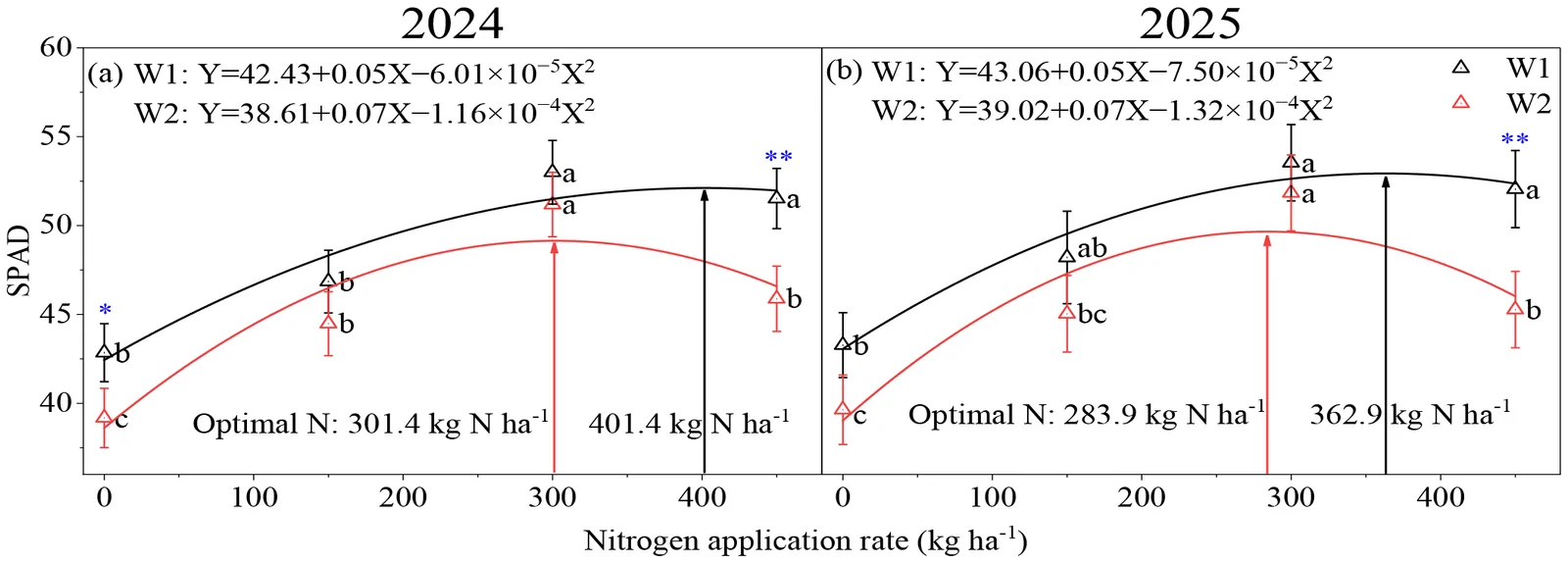
Effects of nitrogen fertilizer application rates on leaf SPAD values under different irrigation levels in 2024 **(a)** and 2025 **(b)**. W1 and W2 represent full irrigation and reduced irrigation, respectively. Different lowercase letters indicate significant differences among treatments within the same irrigation level at the 0.05 level (*P* < 0.05). **indicates significant differences among different irrigation levels under the same nitrogen rate at the 0.01 level (*P* < 0.01).

Irrigation and nitrogen application rate significantly affected the SPAD values (all *P* < 0.001; **Table 1**). Specifically, compared with W1, W2 decreased the leaf SPAD values (**Figure 5**). Under both W1 and W2, the leaf SPAD values exhibited a parabolic trend, initially increasing and then decreasing, as the nitrogen application rate increased. The average optimal nitrogen application rates for leaf SPAD values under W1 and W2 were 382.2 kg ha^-1^ and 292.7 kg ha^-1^, respectively.

### 3.4 Stomatal characteristics and leaf enzyme activity and concentration

**Figure 6:**
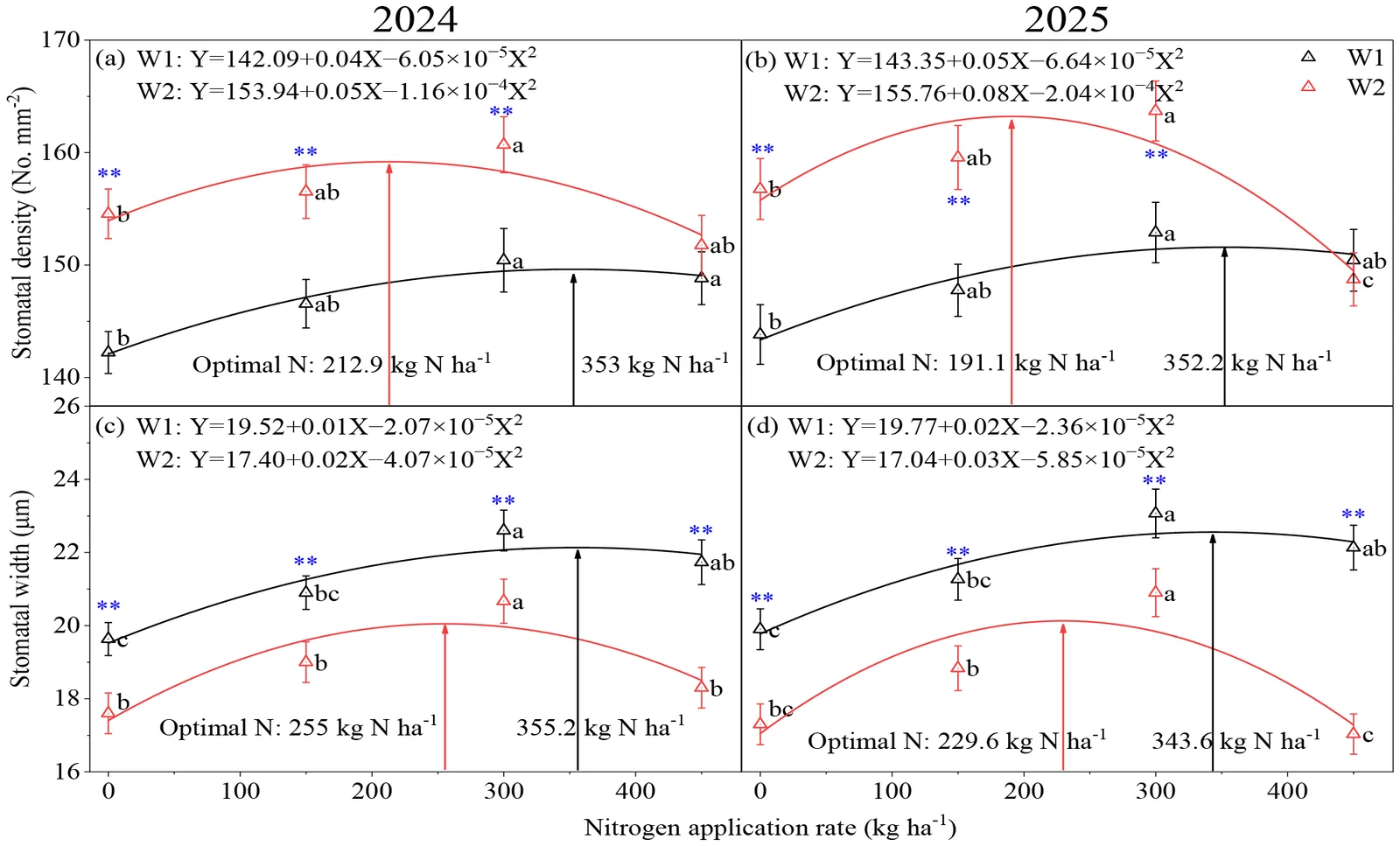
Effects of nitrogen fertilizer application rates on leaf stomatal density **(a, b)** and stomatal width **(c, d)** under different irrigation levels. W1 and W2 represent full irrigation and reduced irrigation, respectively. Different lowercase letters indicate significant differences among treatments within the same irrigation level at the 0.05 level (*P* < 0.05). **indicates significant differences among different irrigation levels under the same nitrogen rate at the 0.01 level (*P* < 0.01).

Irrigation, nitrogen application rate, and their interaction significantly altered the leaf stomatal density and stomatal width (all *P* < 0.001; **Table 1**). Overall, compared with W1, W2 increased the leaf stomatal density, which exhibited a parabolic trend, initially increasing and then decreasing, as the nitrogen application rate increased (**Figure 6**). The average optimal nitrogen application rates for stomatal density under W1 and W2 were 352.6 kg ha^-1^ and 202.0 kg ha^-1^, respectively. Under N0, N1, and N2, W2 significantly increased the stomatal density compared to W1 (all *P* < 0.01). The average optimal nitrogen application rates for stomatal width under W1 and W2 were 349.4 kg ha^-1^ and 242.3 kg ha^-1^, respectively. Across all nitrogen application rates, W2 significantly reduced the stomatal width compared to W1 (all *P* < 0.01).

**Figure 7:**
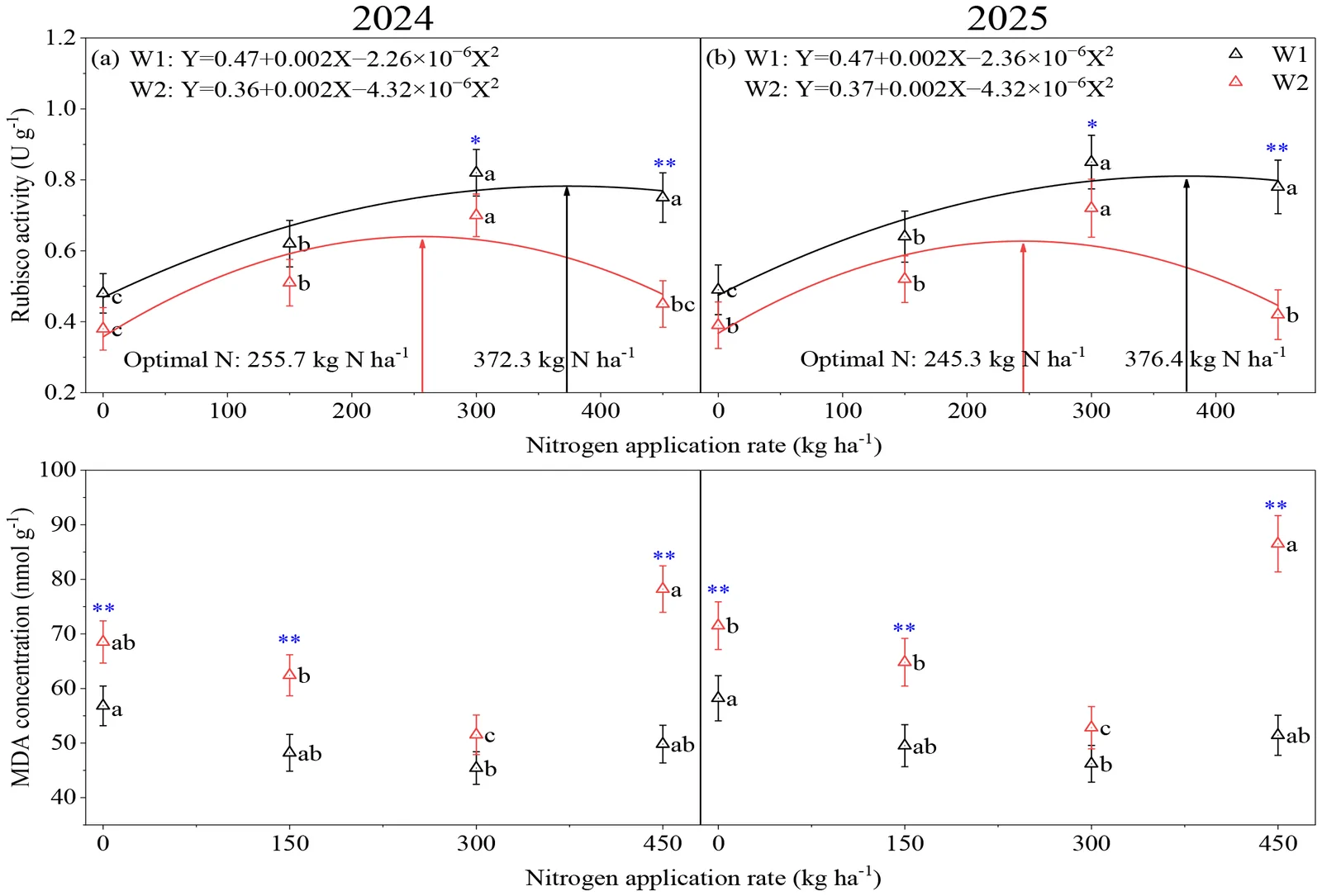
Effects of nitrogen fertilizer application rates on leaf ribulose-1,5-bisphosphate carboxylase/oxygenase (Rubisco) activity **(a, b)** and malondialdehyde (MDA) concentration **(c, d)** under different irrigation levels. W1 and W2 represent full irrigation and reduced irrigation, respectively. Different lowercase letters indicate significant differences among treatments within the same irrigation level at the 0.05 level (*P* < 0.05). * and ** indicate significant differences among irrigation levels under the same nitrogen rate at the 0.05 and 0.01 levels, respectively.

Irrigation, nitrogen application rate, and their interaction significantly affected Rubisco activity and MDA concentration (all *P* < 0.001; **Table 1**). Overall, compared with W1, W2 decreased the Rubisco activity, and the average optimal nitrogen application rates for Rubisco activity under W1 and W2 were 374.4 kg ha^-1^ and 250.5 kg ha^-1^, respectively (**Figure 7**). Specifically, under W1, the N1, N2, and N3 treatments significantly increased the leaf Rubisco activity compared to N0 (all *P* < 0.05); under W2, the N2 treatment significantly increased the Rubisco activity compared to N0 (*P* < 0.001). Furthermore, under N0, N1, and N3, W2 significantly increased the leaf MDA concentration compared to W1, whereas no significant difference was observed under N2 (all *P* < 0.01).

### 3.5 Key factor identification for *P*_n_ based on random forest

**Figure 8:**
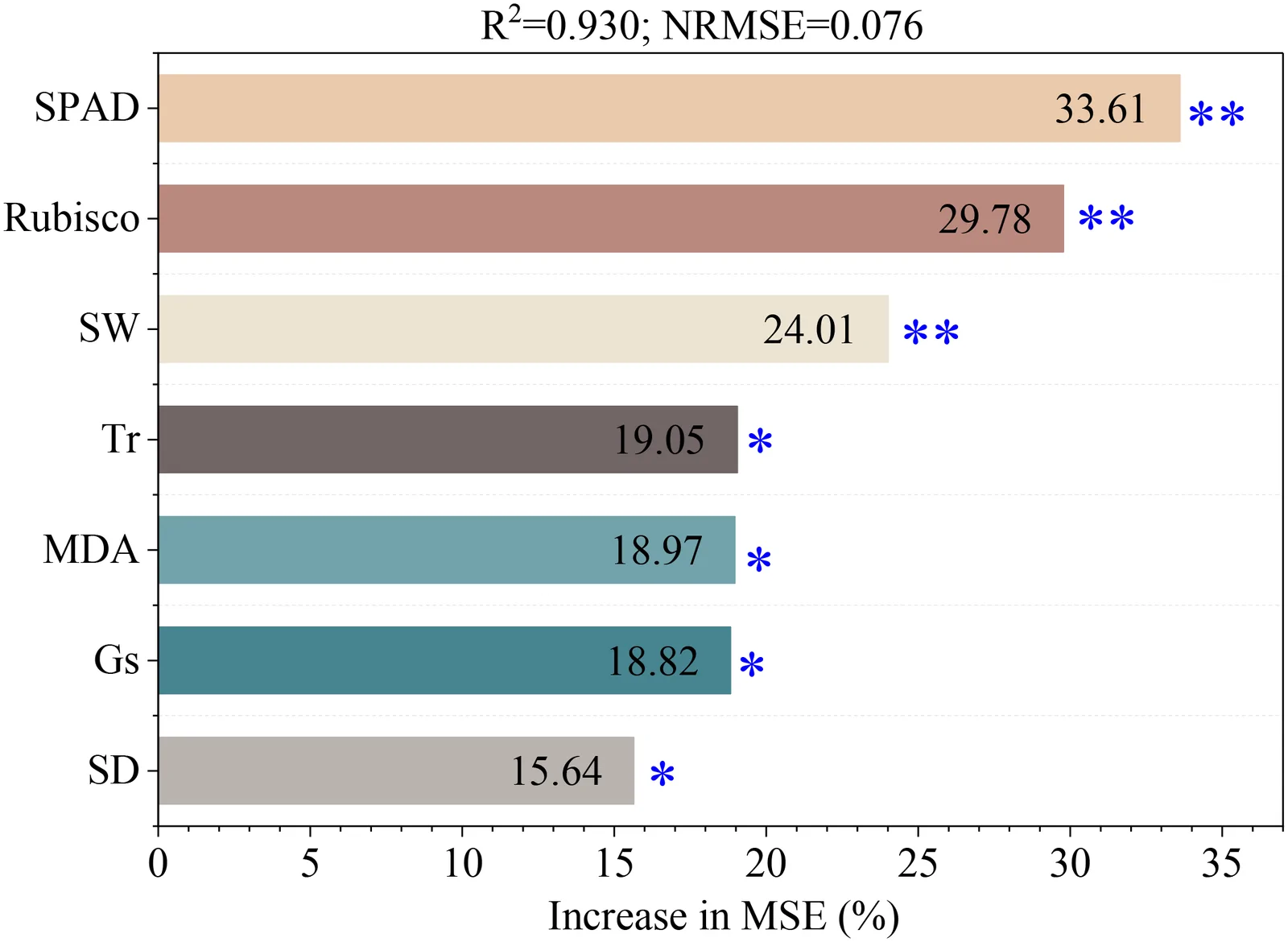
Key physiological factors influencing net photosynthetic rate under water–nitrogen coupling as determined by the random forest model. Rubisco, SW, *T*_r_, *G*_s_, MDA, and SD represent Rubisco activity, stomatal width, transpiration rate, stomatal conductance, MDA concentration, and stomatal density, respectively. * and ** indicate significance at the 0.05 and 0.01 levels, respectively.

To exclusively evaluate the relative importance of micro-physiological variables in explaining the variation of leaf-level net photosynthetic rate (*P*_n_), a random forest model was constructed (**Figure 8**). The study identified seven key physiological indicators, which were SPAD, Rubisco activity, stomatal width, *T*_r_, *G*_s_, MDA, and stomatal density. Their explained variances were 33.61%, 29.78%, 24.01%, 19.05%, 18.97%, 18.82%, and 15.64%, respectively. The total explained variance of these indicators for *P*_n_ was 93.00%.

## 4 Discussion

### 4.1 Appropriate nitrogen application narrows the yield gap and enhances resource use efficiency via the “fertilizer-driven water regulation” effect

In greenhouse agriculture within arid and semi-arid regions, formulating rational water and fertilizer management strategies to balance crop yield and water consumption is central to achieving sustainable development (Li et al., 2017; Yu et al., 2023). This study demonstrates that the responses of tomato fruit yield, aboveground biomass, and water productivity (WP) to the nitrogen application rate all exhibited a significant quadratic parabolic trend. This result verifies our hypothesis (i): excessively increasing nitrogen input does not lead to a limitless increase in yield; rather, there exists a distinct optimal benefit threshold. Notably, under the non-nitrogen (N0) or excessive nitrogen (N3) conditions, reduced irrigation (W2) significantly decreased the fruit yield and aboveground biomass. However, when the nitrogen application rate was at an optimal level (N2), the difference in fruit yield between the W1 and W2 treatments was eliminated. This fully demonstrates that an appropriate nitrogen supply can trigger the “fertilizer-driven water regulation” compensation effect, effectively offsetting the potential yield loss caused by the lowered irrigation limit (65%FC) (Agami et al., 2018; Wang et al., 2022). Furthermore, compared with W1, W2 increased WP (9.9%–11.7%) and agronomic nitrogen use efficiency (ANUE, 30.4%–35.1%). This indicates that a moderate water deficit coupled with an appropriate nitrogen application rate represents an ideal field management strategy for synergistically achieving “stable yield” and “highly efficient use of water and fertilizer.”

Furthermore, W2 induced a leftward shift in the plant’s nitrogen demand threshold, which further supports our scientific hypothesis. Based on the parabolic fitting results, the optimal nitrogen application rates for both macroscopic and microscopic indicators under the W2 treatment were consistently lower than those under full irrigation (W1). This phenomenon may have resulted from the interaction between the decreased soil water content and the elevated fertilizer concentration in the root zone (Wu et al., 2019; He et al., 2023). Under W2, the reduction in total soil water weakened the capacity to dilute fertilizers (Wang et al., 2024). As the nitrogen application rate increased, the lower baseline moisture rapidly drove up the electrical conductivity (EC) of the soil solution (Guo et al., 2017). To avoid severe root osmotic stress and ion stress, the physiological saturation point of nitrogen for the plant’s photosynthetic system and yield formation was reached prematurely, thereby causing the optimal fertilization threshold to shift toward lower nitrogen levels (Liao et al., 2022; Lai et al., 2026).

However, an in-depth comparison of the threshold shift magnitudes across different indicators reveals a phenomenon of “Spatiotemporal Scale Decoupling”: under the W2 condition, the optimal nitrogen application rates for macroscopic indicators reflecting whole-life-cycle cumulative effects (279.9 kg ha^-1^ for yield and 264.8 kg ha^-1^ for WP) were lower than that for microscopic indicators reflecting single-leaf instantaneous physiological activity (e.g., 300.1 kg ha^-1^ for net photosynthetic rate, *P*_n_). The underlying mechanisms driving this pattern of “higher microscopic photosynthetic tolerance, yet earlier macroscopic output saturation” are primarily reflected in the following two aspects: (1) The trade-off between instantaneous assimilation benefits and life-cycle metabolic costs: *P*_n_ is a single-leaf instantaneous physiological indicator measured during the fruit expansion stage. Under the W2 condition, a nitrogen application rate of 300.1 kg ha^-1^ could still provide functional leaves with sufficient nutrients to maintain the maximum instantaneous photosynthetic carbon fixation rate. However, yield and WP are macroscopic endpoint results dynamically accumulated over the entire growth period. Under W2, maintaining a relatively high nitrogen level of 300 kg ha^-1^ throughout the entire period would inevitably increase the whole-plant maintenance respiration and exacerbate long-term implicit osmotic stress costs (Mostafa et al., 2021; Shin et al., 2024). Therefore, from the perspective of comprehensive life-cycle benefits, the system already achieved the ultimate “benefit-cost” balance at a lower nitrogen application rate (265–280 kg ha^-1^). (2) The mechanism of active vegetative growth sacrifice and reallocation based on the source-sink relationship: Although higher nitrogen levels can maintain maximum *P*_n_ at the single-leaf scale, they tend to stimulate competitive consumption of photosynthetic assimilates by stems and leaves (Vos, 1995; Zhu et al., 2024). Macroscopically, reduced irrigation prompted the plant to adopt an allocation strategy that sacrificed vegetative growth to prioritize reproductive growth (She et al., 2017; Jiao et al., 2024). Our experimental data substantiates this conclusion: under the optimal nitrogen application levels (N2), there was no significant difference in fruit yield between the W1 and W2 treatments, yet the aboveground biomass of W2 was significantly lower than that of W1. This indicates that under a moderate water deficit, the plant actively suppressed redundant stem and leaf expansion, more efficiently reallocating the limited photosynthetic assimilates toward the fruits (the “sink”) (Jiao et al., 2024). Attributable to this higher harvest index and source-sink reallocation efficiency, the fruit yield could fully peak at a relatively lower nitrogen application rate (279.9 kg ha^-1^). This explains why the W2 treatment could achieve equivalent yield compensation and increased water productivity with lower aboveground construction costs and fertilizer inputs.

### 4.2 Compensatory mechanism of carbon assimilation synergistically driven by stomatal morphological optimization and Rubisco activity maintenance

Elucidating how water and fertilizer management affects the final yield through microscopic physiological processes is the core objective of this study. The results of the random forest model supported hypothesis (ii), indicating that the leaf SPAD value, intrinsic water use efficiency (*WUE*_i_), Rubisco activity, and stomatal width are the most critical driving factors determining the net photosynthetic rate, with a cumulative explained variance exceeding 80%. As the portals regulating leaf gas exchange, plant stomata, specifically their development and opening/closing strategies, are highly sensitive to the water-nitrogen interactive environment (Zheng et al., 2020). This study revealed that the W2 treatment increased the leaf stomatal density, while simultaneously decreasing the stomatal width across all nitrogen application levels. This morphological remodeling toward a “high-density, small-aperture” configuration is a classic optimization strategy for plants to adapt to drought stress (Liu et al., 2024). On the one hand, smaller stomatal guard cells possess a faster rate of turgor regulation, which can significantly accelerate the dynamic response of stomatal opening and closing, enabling plants to withstand external environmental fluctuations and stress more rapidly and flexibly (Caine et al., 2023; Zhang et al., 2026). On the other hand, this morphological shift restricts excessive overall water loss (manifested as the significant reductions in *T*_r_ and *G*_s_) while maintaining essential carbon dioxide influx pathways relying on the high-density stomatal distribution (Zheng et al., 2020). Benefiting from this precise and highly sensitive stomatal regulation mechanism, *WUE*_i_ under W2 surpassed that under W1 at nitrogen application rates exceeding 300 kg ha^-1^.

Apart from the physical resistance of stomata, the biochemical carbon fixation capacity represented by Rubisco activity plays an equally decisive role (Zhuang et al., 2020; Suganami et al., 2021). This study elucidates the interactive effects of water and fertilizer on the biochemical enzyme activity within mesophyll cells: although the W2 treatment overall reduced Rubisco activity, the Rubisco activity under the moderate nitrogen application rates (N1 and N2) exhibited a significant stimulation and enhancement compared with the non-nitrogen control (N0). The maintenance of this critical photosynthetic enzyme activity perfectly compensated for the substrate supply limitation caused by the reduction in stomatal conductance, thereby ensuring that the leaves could sustain a high carbon assimilation capacity at a lower transpiration cost. This dual optimization of both the morphological (stomatal development) and biochemical (Rubisco activity) pathways collectively constitutes the physiological mechanism underlying the narrowed yield gap achieved under the appropriate nitrogen application rate.

### 4.3 Biochemical mechanisms of compounded stress and photosynthetic impairment induced by excessive nitrogen application

This study further reveals the mechanism of physiological toxicity caused by the blind application of excessive nitrogen under restricted water conditions, providing direct evidence for our hypothesis (iii). When reduced irrigation was combined with a high nitrogen application rate (W2 + N3), it not only failed to further promote crop growth but conversely led to severe impairment of the photosynthetic system (Liao et al., 2022). This physiological decline may be corroborated by the indicator of cellular oxidative damage: under the N0, N1, and excessive nitrogen (N3) levels, the W2 treatment significantly increased the malondialdehyde (MDA) concentration in leaves compared to W1. The rapid accumulation of MDA is a direct hallmark of severe oxidative damage to the cellular plasma membrane system (Wu et al., 2023). Under limited soil moisture conditions, a fertilizer input as high as 450 kg ha^-1^ could decreases the osmotic potential of the soil solution, subjecting the root system to a severe “compounded stress” of salinity and drought.

This intense compounded stress may not only physically force further stomatal closure (a decrease in stomatal width) but also potentially disrupt the stability of chloroplast structures at the biochemical level, which is manifested as a significant decline in SPAD values after exceeding the optimal nitrogen threshold (**Figure 5**). The intracellular accumulation of reactive oxygen species (ROS) and the damage to the membrane system ultimately led to the inactivation of Rubisco, triggering a sharp decline in *P*_n_ and a significant reduction in the final yield (Wang et al., 2015). However, under the optimal nitrogen application level (N2), there was no significant difference in MDA concentration between the W1 and W2 treatments. This contrast provides evidence that a precisely optimal nitrogen input under reduced irrigation not only averts physiological harm but also effectively maintains the balance of the plant’s antioxidant system and protects the integrity of the cellular membrane structure (Chen et al., 2013). Therefore, strictly controlling the upper limit of nitrogen application under reduced irrigation to avoid compounded stress induced by water-fertilizer antagonism is a critical prerequisite for achieving both water conservation and high yields in greenhouse tomatoes (Li et al., 2017; Sun et al., 2024).

From the perspective of agronomic practice and sustainable production, in a greenhouse environment shielded from precipitation interference, pinpointing the microscopic physiological critical threshold where nitrogen shifts from “stress resistance compensation” to “osmotic stress” is of significant value for guiding precise resource management in intensive agriculture within arid regions. This helps propel the integrated water and fertilizer management in greenhouse facilities from a traditional “experience-driven” approach to a “data-driven” paradigm based on the real-time physiological demands of crops. However, water conservation, yield preservation, and efficient carbon fixation in crops constitute a systemic process encompassing the “soil-root-aboveground” continuum. Although this study elucidated the macroscopic effects of water-fertilizer antagonism from the microscopic scales of leaf stomata, photosynthetic enzyme activity, and cellular lipid peroxidation, it has not yet conducted direct observations of the belowground root architecture and fine-scale changes in the root-zone soil microenvironment (Zhu et al., 2025). Therefore, future research should further incorporate long-term *in-situ* greenhouse monitoring to deeply decipher the underlying driving mechanisms by which reduced irrigation combined with different nitrogen application rates affects the evolution of secondary soil salinization in the root zone, the development of spatial root architecture, and root-sourced signal transduction pathways. These investigations will provide a more systematic and robust theoretical foundation for the sustainable intensification of greenhouse agriculture in arid and semi-arid regions from the holistic scale of aboveground-belowground synergistic regulation.

## 5 Conclusion

This study systematically evaluated the interactive effects of irrigation and nitrogen on greenhouse tomatoes. The main conclusions are: (1) Reduced irrigation (W2, 65% FC) generally decreased fruit yield. However, coupling W2 with an appropriate nitrogen supply eliminated the yield gap compared to full irrigation (W1), while significantly increasing water productivity (WP) by 9.9%–11.7% and agronomic nitrogen use efficiency by 30.4%–35.1%. (2) Reduced irrigation shifted the optimal nitrogen requirements toward lower levels. Based on quadratic regression, the estimated optimal nitrogen application rates for maximizing fruit yield and WP under W2 were 279.9 and 264.8 kg ha^-1^, respectively. (3) Physiologically, optimal nitrogen under W2 maintained high leaf carbon assimilation by inducing “high-density, small-aperture” stomatal morphological configuration and sustaining Rubisco activity. Conversely, excessive nitrogen (450 kg ha^-1^) under water deficit caused severe oxidative damage (MDA accumulation) and degraded the photosynthetic capacity. In conclusion, setting the irrigation lower limit to 65% FC combined with a precise nitrogen application rate of 265–280 kg ha^-1^ is the recommended management strategy to synergistically achieve stable yields and high water-fertilizer use efficiency.

## Data Availability

The raw data supporting the conclusions of this article will be made available by the authors, without undue reservation.
